# Localizing the 5Cs Scale in China: when teacher credibility outperforms confirmation in high-stakes thesis writing

**DOI:** 10.3389/fpsyg.2026.1955402

**Published:** 2026-10-20

**Authors:** Yuan Sun, Guanghui He, Zhaolong Niu

**Affiliations:** 1 Zhengzhou Shengda University of Economic Business and Management, Zhengzhou, China; 2 School of Nuclear Science and Technology, Lanzhou University, Lanzhou, China

**Keywords:** 5Cs Scale, context-dependency, learner empowerment, positive psychology, teacher credibility, thesis writing

## Abstract

This study localizes the Iranian-developed 5Cs Scale within the Chinese context and examines how positive psychology-informed teaching behaviors are associated with English major undergraduates’ empowerment in high-cognitive-load thesis writing courses. Through translation, back-translation, and cultural adaptation, a 49-item Chinese version of the 5Cs Scale was developed and administered to 446 third-year English majors from two universities in China. Reliability, validity, and structural equation modeling analyses were performed. The scale demonstrated excellent reliability and sound structural validity. The findings reveal a differentiated pattern of statistical associations: teacher credibility showed the strongest association with perceived course impact; teacher care showed the strongest association with perceived course meaningfulness; and teaching clarity showed the strongest association with perceived course competence. Notably, in the context of Chinese thesis writing, teacher credibility emerged as the strongest and most consistent statistical predictor across all three empowerment dimensions—a pattern that contrasts with previous findings from the Iranian EFL context, where confirmation was the strongest predictor. The localized 5Cs Scale offers a valid instrument for future research, and the findings—particularly the difference in the relative predictive strength from confirmation to credibility—highlight the context-dependent nature of positive teacher interpersonal behaviors and warrant further longitudinal and experimental investigation.

## 1 Introduction

Educational researchers widely recognize teachers as “central agents in any learner’s educational journey” (Mercer and Dörnyei, 2020, p. 72). In the classroom context, the teacher is regarded as the empowering and the student as the empowered (Frymier and Houser, 1999). High-quality teacher-student relationships significantly enhance students’ motivation, engagement, and psychological well-being (Roorda et al., 2011). However, for Chinese English majors, the function of this relationship faces particular challenges in the context of thesis writing—a task characterized by high cognitive load and compounded by high stakes, as the outcome directly affects students’ graduation and academic standing.

Thesis writing for Chinese English majors is a typical high-cognitive-load task. Academic writing challenges, including difficulties with grammar, logical organization, and expressing complex ideas, have been documented among doctoral students (Gupta et al., 2022). These challenges are not confined to the doctoral level; undergraduate thesis writers face comparable obstacles when producing extended academic texts under high-stakes conditions. Students are required to simultaneously mobilize language proficiency, critical thinking, knowledge of academic conventions, and self-regulation skills when selecting topics, reviewing literature, constructing theoretical frameworks, organizing logical arguments, and expressing ideas in academic English (Sweller, 1988). The complexity of this task often exceeds students’ available cognitive resources, leading to pervasive writing anxiety, self-doubt, and even procrastination. However, traditional thesis writing instruction has focused primarily on skill transmission and adherence to norms, largely neglecting students’ emotional experiences and psychological needs in high-load tasks. Purely cognitive guidance not only fails to alleviate students’ anxiety, but may further exacerbate cognitive overload by adding “pressure to be correct”.

The introduction of Positive Psychology (PP) offers a new perspective for addressing this dilemma. Unlike traditional psychology, which focuses on problems and deficits, PP aims to cultivate individuals’ positive emotions, character strengths, and supportive environments (Seligman and Csikszentmihalyi, 2000). In educational settings, teachers’ positive interpersonal behaviors—such as showing care, providing clear instruction, building rapport, offering confirmation, and demonstrating credibility—can satisfy students’ basic psychological needs for autonomy, competence, and relatedness, thereby enhancing their intrinsic motivation and psychological resilience (Deci and Ryan, 2000). Particularly in high-cognitive-load situations, positive emotions have a “broaden-and-build” function (Fredrickson, 2001), helping students disengage from anxiety and more effectively allocate cognitive resources to complex writing tasks.

Therefore, investigating how positive-psychology-informed teacher interpersonal behaviors empower students—that is, enhance their perceived course impact, meaningfulness, and competence—in the specific context of English major thesis writing (characterized by high cognitive load, high stakes, and high evaluative anxiety) carries unique theoretical value and practical urgency. Existing research has validated the benefits of positive psychology in general EFL classrooms, yet its application in high-cognitive-load instructional settings remains largely unexplored. The present study aims to fill this gap by localizing a relevant measurement tool and, through empirical investigation, revealing the pathways through which teacher interpersonal behaviors influence student empowerment in the Chinese cultural context, thereby providing empirical evidence for optimizing thesis writing instruction and strengthening students’ psychological resources. To this end, this study adopts a two-stage design. Stage 1 focuses on the localization and psychometric validation of the 5Cs Scale in the Chinese EFL context. Stage 2, building on the validated instrument, tests a structural model linking five teacher interpersonal behaviors to three dimensions of learner empowerment.

## 2 Literature review

### 2.1 Positive psychology in L2 education and thesis writing

Positive Psychology (PP) emerged as a paradigm shift in psychology at the turn of the 21st century, moving the field’s focus from repairing pathology and deficits to cultivating human strengths, well-being, and optimal functioning (Seligman and Csikszentmihalyi, 2000). In educational contexts, PP emphasizes the exploration and development of learners’ positive emotions, character strengths, such as resilience, optimism, gratitude, hope, and supportive learning environments. The broaden-and-build theory (Fredrickson, 2001) is central to PP: positive emotions broaden individuals’ momentary thought-action repertoires, enabling them to see more possibilities, think more flexibly, and build enduring personal resources, e.g., coping strategies, social connections, resilience over time.

The application of PP to L2 education gained momentum after the seminal special issue of Studies in Second Language Learning and Teaching (MacIntyre and Mercer, 2014) and subsequent edited volumes (Gabryś-Barker and Gałajda, 2016). Researchers have since investigated a range of positive variables, including foreign language enjoyment (Dewaele et al., 2018; Dewaele and MacIntyre, 2022), grit (Teimouri et al., 2020), resilience (Wang et al., 2021), and growth mindset (Lou and Noels, 2020). These studies have generally confirmed that positive emotions and traits enhance L2 learning outcomes by increasing motivation, engagement, and persistence. More recently, the field has witnessed an integrative turn from traditional positive psychology to existential positive psychology (EPP) and PP 2.0, which emphasize that learner well-being and ill-being are intertwined, and that both positive and negative emotions should be examined together in context (Derakhshan and MacIntyre, 2026). Recent research has further extended this line of inquiry to technology-mediated contexts, showing that AI-enhanced language tools are associated not only with higher grit and enjoyment but also with reduced anxiety and emotional disengagement among multilingual learners (Wang, 2026).

In the specific domain of L2 writing, PP research has gained traction but remains limited. Studies have examined learners’ emotional experiences in L2 writing (Zare et al., 2023), self-regulatory processes that underpin writing confidence (Teng and Zhang, 2022), and the role of teacher interpersonal behaviors in shaping students’ writing-related emotions (Dewaele and Li, 2021; Pearson, 2024). However, the majority of these studies have been conducted in low-to-moderate cognitive load settings, such as routine composition classes or short essay tasks. Thesis writing—a genre characterized by extended length, original argumentation, literature synthesis, and adherence to academic conventions—imposes an exceptionally high cognitive load (Sweller, 1988). Students must simultaneously manage multiple sub-tasks: identifying research gaps, organizing literature, constructing theoretical frameworks, maintaining logical coherence, and using discipline-appropriate language. This complexity often exceeds students’ available cognitive resources, leading to writing anxiety, self-doubt, procrastination, and even avoidance. The high cognitive load is further amplified by the high-stakes nature of the task, as thesis grades often determine graduation and degree classification.

Traditional writing instruction has focused primarily on skill transmission (e.g., grammar correction, citation formatting) and cognitive strategies (e.g., outlining, drafting). While necessary, this approach largely neglects students’ affective and motivational experiences in high-load tasks. Purely cognitive guidance may inadvertently increase students’ “pressure to be correct” and exacerbate cognitive overload rather than alleviate it. Therefore, it is both theoretically and practically urgent to investigate how positive psychology principles—such as fostering positive emotions, satisfying basic psychological needs, and building supportive teacher-student relationships—can empower students in high-cognitive-load thesis writing contexts.

### 2.2 Teacher interpersonal behaviors: from general pedagogy to the 5Cs framework

A substantial body of instructional communication research has demonstrated that teacher interpersonal behaviors are among the strongest predictors of student motivation, engagement, and learning outcomes (Houser and Hosek, 2018; Frymier et al., 2019). The rhetorical/relational goal theory (Mottet et al., 2006) provides a unifying framework: in the classroom, teachers pursue both rhetorical goals (i.e., communicating content clearly, demonstrating competence) and relational goals (i.e., building rapport, showing care, confirming students’ worth). When teachers successfully fulfill both sets of goals, students experience greater satisfaction, motivation, and learning.

Research consistently shows that positive teacher interpersonal communication behaviors boost students’ academic engagement, motivation, achievement, and psychological well-being. Teacher care fosters security and self-esteem (Gasser et al., 2018; Derakhshan et al., 2019); teacher clarity facilitates information processing and learning (Bolkan, 2017; Titsworth et al., 2015; Violanti et al., 2018); teacher confirmation enhances motivation and satisfaction (Ellis, 2000; Goodboy et al., 2009; Goldman et al., 2014); teacher credibility predicts attendance and achievement (Teven and McCroskey, 1997; Gray et al., 2011; Pishghadam et al., 2021); teacher immediacy reduces anxiety and boosts engagement (Ballester, 2015; Frymier et al., 2019); teacher stroke improves motivation and success (Pishghadam and Khajavy, 2014; Rajabnejad et al., 2017); and teacher-student rapport fosters participation and autonomy (Wilson and Ryan, 2013; Culpeper and Kan, 2020). While these interpersonal behaviors have been extensively validated in traditional settings, their roles may need to be re-examined in technology-mediated environments. Recent studies in AI-assisted EFL contexts suggest that students differentially value affective, pedagogical, technical, autonomy, and feedback support (Wang et al., 2025), and that teacher immediacy and rapport remain critical for learners’ willingness to communicate (Zhi and Wang, 2024). These findings highlight the need to examine how teacher interpersonal behaviors operate in specific high-stakes contexts, such as thesis writing. Collectively, these established effects align with the rhetorical and relational goal theory (Mottet et al., 2006; Houser and Hosek, 2018). To systematically measure positive teacher interpersonal behaviors, Derakhshan (2022) further proposed the 5Cs framework, which integrates five empirically validated dimensions: Care, Clarity, Closeness, Confirmation and Credibility.

Care refers to teachers’ verbal and nonverbal actions that demonstrate recognition of and responsiveness to students’ psychological, emotional, and academic needs (Noddings, 1984). In the classroom, care is manifested through encouragement, attentiveness, providing help, and showing empathy. Teacher care has been shown to predict student engagement, willingness to communicate, and reduced academic burnout (Derakhshan, 2022; Pishghadam et al., 2021).

Clarity pertains to the logical organization, comprehensibility, and transparency of instruction (Chesebro and McCroskey, 1998). Clear teachers define major concepts explicitly, use relevant examples, provide unambiguous assignment guidelines, and answer questions effectively. Clarity reduces students’ cognitive load, facilitates information processing, and predicts affective and cognitive learning (Bolkan et al., 2015; Titsworth et al., 2015).

Closeness captures the degree of emotional bonding, warmth, and trust in the teacher-student relationship (Derakhshan, 2022). Close teacher-student relationships are characterized by mutual respect, low conflict, and open communication. Closeness has been linked to student engagement, motivation, reduced classroom anxiety, and teaching effectiveness (Roorda et al., 2011; Leng and Zhang, 2025).

Confirmation refers to teachers’ communicative behaviors that make students feel valued, significant, and endorsed (Ellis, 2000). Confirmation comprises three components: responding to students’ questions, demonstrating interest in students’ learning, and using an interactive teaching style. Teacher confirmation positively predicts student motivation, affective learning, and cognitive learning (Goodboy et al., 2009; Goldman et al., 2014).

Credibility is students’ perception of the teacher as competent, trustworthy, and possessing goodwill toward students (McCroskey and Teven, 1999). Credibility includes three sub-dimensions: competence (knowledge and expertise), trustworthiness (honesty and reliability), and goodwill (genuine care for students’ interests). Credible teachers are more likely to be listened to, respected, and emulated by students, and credibility has been shown to predict student motivation, affective learning, and cognitive learning (Finn et al., 2009; Zhang, 2009).

In the Iranian EFL university context, Derakhshan (2022) validated the full 5Cs scale and tested a structural model in which the five teacher behaviors predict learner empowerment, which in turn predicts affective, behavioral, and cognitive learning. The results indicated that all five 5Cs dimensions significantly predicted empowerment, with confirmation emerging as the strongest predictor (β = 0.29) and credibility the weakest (β = 0.14). Learner empowerment also mediated the effects of 5Cs behaviors on learning outcomes. These findings provide a direct point of comparison for the present study.

Despite these robust findings, the 5Cs framework has not yet been empirically tested in other cultural contexts—particularly in Chinese English major thesis writing courses. Cultural differences between Iran and China may affect how students perceive and respond to each 5Cs dimension, and the high-cognitive-load nature of thesis writing may further alter the relative predictive power of the five behaviors. For instance, in lower-load general EFL classrooms, confirmation and care may be paramount; in high-load, high-stakes writing tasks, students may prioritize credibility because they need to trust their teacher’s expertise to guide them through uncertainty. These possibilities await empirical investigation.

### 2.3 Learner empowerment: conceptualization and antecedents

Learner empowerment originates from organizational psychology and was adapted to educational contexts by Frymier et al. (1996). They conceptualized learner empowerment as a motivational state comprising three dimensions. (a) Impact: the student’s belief that they can make a difference in the course, influencing what happens, having a voice, and affecting outcomes; (b) Meaningfulness: the student’s perception that the course content, tasks, and activities are valuable, interesting, and personally relevant; and (c) Competence: the student’s confidence in their ability to perform required tasks successfully and achieve course goals. Empowerment is not simply a personality trait; it is state-like and context-dependent, heavily influenced by the quality of teacher-student interpersonal relationships (Frymier et al., 1996; Houser and Frymier, 2009). When students feel empowered, they are more likely to engage deeply, persist through challenges, and take ownership of their learning. Recent research further suggests that such psychological resources are critical for success in demanding academic writing tasks, with learner engagement and grit predicting EFL writing achievement (Li and Yuan, 2025). Consequently, understanding how teacher behaviors can foster these empowering psychological states becomes essential.

Previous research has identified several teacher behaviors as antecedents of learner empowerment. Teacher clarity and nonverbal immediacy predict all three empowerment dimensions (Finn and Schrodt, 2012). Teacher confirmation has been shown to positively relate to student empowerment (Goldman et al., 2014). Teacher credibility, especially the competence dimension, is associated with students’ sense of influence and meaningfulness (Schrodt et al., 2008). In the Iranian EFL context, Derakhshan (2022) found that all five 5Cs behaviors significantly predicted empowerment, with confirmation being the strongest predictor and credibility the weakest.

Crucially, whether the three dimensions of empowerment are differentially influenced by specific teacher behaviors has not been empirically tested in a high-cognitive-load academic writing context. The present study aims to fill this gap.

### 2.4 Research hypotheses

Based on the theoretical framework and empirical evidence reviewed above, the present study proposes the following hypotheses. The theoretical and empirical rationale for the following hypotheses is provided in Sections 2.2 and 2.3. The present study proposes 15 directional hypotheses linking five teacher interpersonal behaviors to three dimensions of learner empowerment.

Teacher care:

H1: Teacher care positively predicts perceived course impact.

H2: Teacher care positively predicts perceived course meaningfulness.

H3: Teacher care positively predicts perceived course competence.

Teacher clarity:

H4: Teacher clarity positively predicts perceived course impact.

H5: Teacher clarity positively predicts perceived course meaningfulness.

H6: Teacher clarity positively predicts perceived course competence.

Teacher-student closeness:

H7: Teacher-student closeness positively predicts perceived course impact.

H8: Teacher-student closeness positively predicts perceived course meaningfulness.

H9: Teacher-student closeness positively predicts perceived course competence.

Teacher confirmation:

H10: Teacher confirmation positively predicts perceived course impact.

H11: Teacher confirmation positively predicts perceived course meaningfulness.

H12: Teacher confirmation positively predicts perceived course competence.

Teacher credibility:

H13: Teacher credibility positively predicts perceived course impact.

H14: Teacher credibility positively predicts perceived course meaningfulness.

H15: Teacher credibility positively predicts perceived course competence.

These hypotheses constitute the conceptual model tested in the present study. The structural model and the results of hypothesis testing are reported in Section 4.5 (see Figure 1).

**Figure 1:**
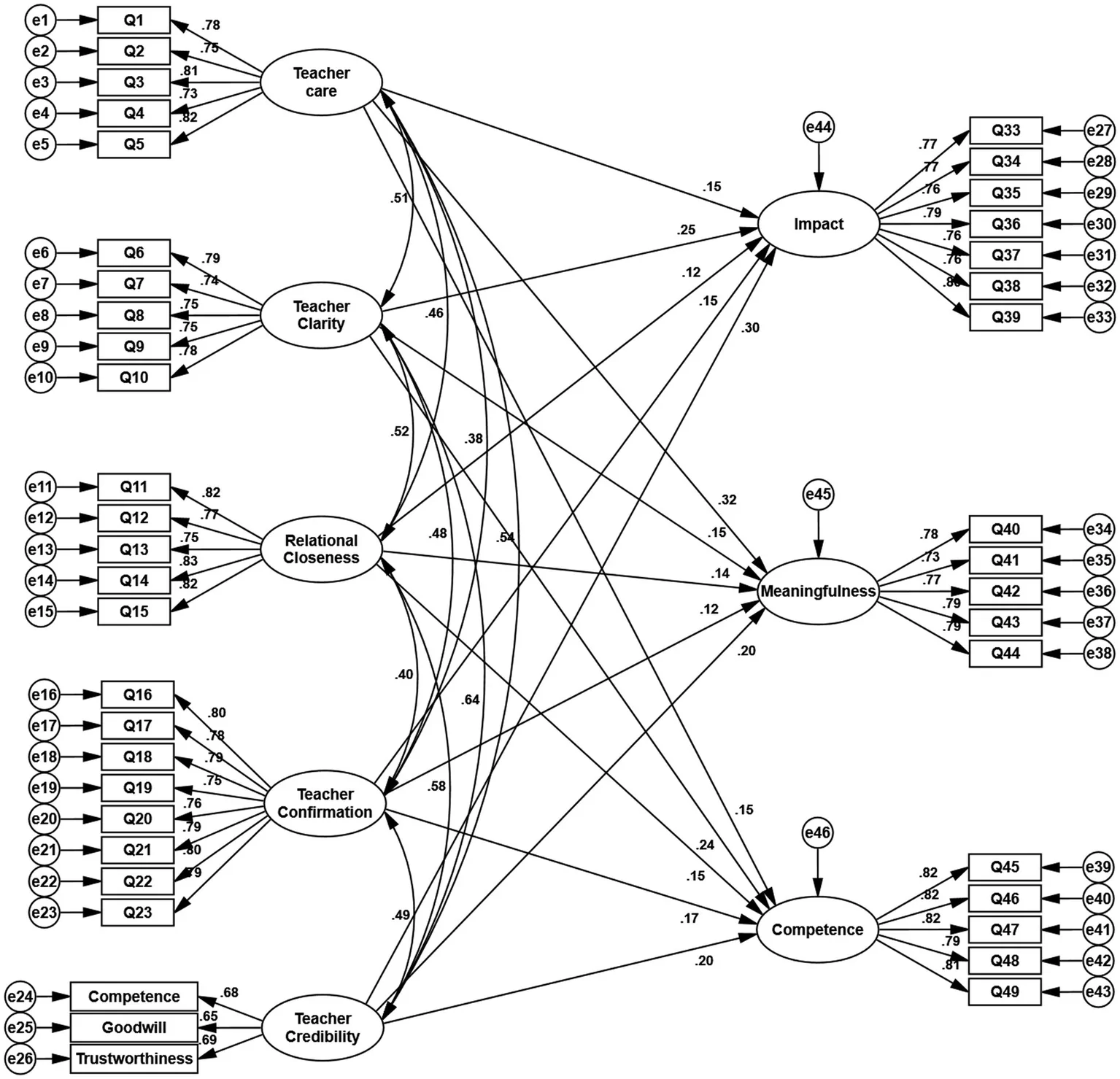
Results of structural equation model analysis (solid lines indicate significant paths; *p < 0.05, **p < 0.01, ***p < 0.001).

### 2.5 The present study

Three gaps emerge from the literature reviewed above. First, positive psychology and teacher interpersonal behavior research have largely focused on general L2 classrooms or low-stakes writing tasks, while high-cognitive-load, high-stakes thesis writing remains underexplored. Second, the 5Cs Scale, originally validated in the Iranian EFL context, has not been systematically localized for Chinese English major undergraduates. Third, while previous studies have examined the overall predictive power of 5Cs behaviors on empowerment, few have investigated differential pathways—that is, whether specific teacher behaviors uniquely predict specific empowerment dimensions.

To address these gaps, the present study has two interconnected objectives: (1) to localize and validate the 5Cs Scale for Chinese English major undergraduates; and (2) to examine how the five teacher behaviors predict the three empowerment dimensions using the validated scale. In Stage 1, the original scale will be adapted through translation, back-translation, cultural adaptation, and expert content validation, resulting in a Chinese version with sound psychometric properties (reliability, construct validity, discriminant validity). In Stage 2, the validated instrument will be used to empirically investigate how five teacher interpersonal behaviors (care, clarity, closeness, confirmation, credibility) predict three dimensions of learner empowerment (impact, meaningfulness, competence) in the context of English major thesis writing courses in China, guided by positive psychology and rhetorical/relational goal theory.

By achieving these objectives, the study will provide a validated measurement tool for future research and offer evidence-based insights into which teacher behaviors most effectively empower students facing high-cognitive-load academic writing tasks—ultimately informing instructional design, teacher training, and student support strategies in Chinese EFL and broader academic writing contexts.

## 3 Methodology

### 3.1 Research participants

This study employed a questionnaire survey targeting undergraduate English majors enrolled in the English thesis writing course. As this course is exclusively offered to third-year students, participants were juniors from two comprehensive universities in China: one key university in western China, and one provincial university in central China. The thesis writing course is a professional compulsory course meeting once every week with each session consisting of two class hours. Instruction addressed various challenges in thesis writing: topic selection, proposal defense, literature review, thesis structure, theoretical application, textual analysis, and academic language expression.

In the formal data collection phase, 500 questionnaires were distributed online and 446 collected all valid with a 89.2% response rate. Participants ranged in age from 20 to 22 years, including 215 males (48.21%) and 231 females (51.79%). Regarding English proficiency, most of the participants had passed the Test for English Majors Band 4 or College English Test Band 4. All participants provided informed consent prior to their participation, and anonymity and confidentiality of the data were strictly maintained throughout the research process.

### 3.2 Research tools: localization of the 5Cs scale

This study adapted the 5Cs Scale synthesized by Derakhshan (2022) in his work *the 5Cs Positive Teacher Interpersonal Behaviors*. The original instrument consists of three sets of scales with a total of 133 items: a 67-item 5Cs scale, a 35-item Learner Empowerment Scale, and a 31-item Learning Effect Scale (measuring affective, behavioral, and cognitive learning). Administering all 133 items in an online survey is impractical. First, the primary focus of this study is to examine how positive teacher interpersonal behaviors empower English majors in their thesis writing courses; therefore, the Learning Effect Scale is not relevant to the research objectives. Second, even the remaining 102 items would risk participants’ fatigue and undermine data quality. Therefore, a reduction and localization of the original questionnaire was necessary.

Five experts were invited to the adaptation process. The experts included three professors in English education (two with PhDs in Applied Linguistics) and two associate professors in educational psychology (both with PhDs). All had prior experience in scale development and cross-cultural adaptation. First, a strict translation-back-translation process followed the Brislin translation model. Two bilingual experts independently translated the original English scale into Chinese. Second, another two bilingual experts, unfamiliar with the scale, back-translated the Chinese version into English. The original and back-translated versions were compared; ambiguous or inconsistent items were discussed and revised. Finally, all five experts conducted cultural adaptation and content validity evaluation.

Necessary modifications were made to ensure cultural and contextual appropriateness. Some items were removed and others reworded. For example, the item “Competence bright-stupid” was removed to align with Chinese classroom discourse norms, as most Chinese college students would not be prone to evaluate their teachers this way. Items with overlapping or redundant meanings were also removed, e.g., the first item “my teacher pays attention to me” in the original Teacher Care Scale was removed because its content is covered by the following eight items. The fourth item in the original questionnaire “my teacher uses my knowledge in the class” was reworded to “my teacher adopts my view on the thesis in class” to better fit the thesis writing course scenario. For credibility items, the concept of “goodwill” was specified with concrete behaviors such as “my teacher concerns my academic needs” to make it more tangible in the Chinese learning context.

Experts rated each item on a 4-point scale (1 = not relevant, 4 = highly relevant). The Item-level Content Validity Index (I-CVI) ranged from 0.80 to 1.00, and the Scale-level CVI/Average (S-CVI/Ave) was 0.94. Disagreements on items were resolved through expert panel discussions until consensus was reached. Based on expert feedback, 50 items from the original 102 were deleted due to cultural or learning context irrelevance. To ensure transparency in the adaptation process, a detailed mapping of the original 102 items to the retained 49 items is provided in Appendix A. The deletion criteria were as follows: (a) items deemed culturally inappropriate for the Chinese educational context (e.g., items containing direct evaluative labels such as ‘bright-stupid’); (b) items with substantive semantic overlap with other items in the same subscale; (c) items that were contextually irrelevant to the specific setting of thesis writing courses; and (d) items with low factor loadings (< 0.50) or poor discrimination indices (critical ratio < 3.00) identified in the pilot study. This systematic deletion process ensured that the retained items adequately cover the conceptual breadth of each construct while maintaining parsimony and cultural relevance.

After the localization and pilot item analysis (see Section 3.3 below), the final version of the 5Cs Scale used in the formal survey consisted of 49 items (see Appendix B) across 8 constructs: a 31-item 5Cs Teacher Interpersonal Behaviors subscale and an 18-item Learner Empowerment subscale. A 7-point Likert scale (1 = Strongly Disagree to 7 = Strongly Agree) was adopted to measure students’ perceptions of teacher behaviors and their own empowerment.

### 3.3 Research procedure and data analysis

Data collection involved two phases: a pilot study (N = 50) to examine item quality and comprehensibility, followed by a formal survey (N = 446) where participants completed the revised online questionnaire midway through the course.

Data analysis was conducted using SPSS 26.0 and AMOS 24.0, involving descriptive statistics, reliability analysis, exploratory factor analysis (EFA), and correlation analysis. The specific procedures were as follows:

1. Item Analysis: During the pilot study, the critical ratio method was employed. All items were ranked by score from lowest to highest. Participants in the top 27% were assigned to the high-score group, while those in the bottom 27% formed the low-score group. Independent samples t-tests were conducted to analyze the differences between these groups for each item, thereby assessing item discrimination. Items that failed to meet the discrimination criteria were removed to form the final scale. Based on this analysis, all items demonstrated acceptable discrimination (*p* < 0.05). However, three items were deleted due to low factor loadings in the exploratory factor analysis of the pilot data: Item 9 (a reverse-coded item), original Item 17, and original Item 40. Consequently, the final scale used in the formal survey contained 49 items.
2. Validity Testing: Content validity was assessed through expert panel review as described in Section 3.2, not repeated here. For construct validity, the formal survey sample was randomly split into two halves. One half was used for Exploratory Factor Analysis (EFA) to explore the internal factor structure; the other half was used for Confirmatory Factor Analysis (CFA) employing Structural Equation Modeling (SEM) to verify the factor structure. Model fit was assessed using χ^2^ /df, RMSEA, IFI, TLI, CFI, and PNFI.
3. Reliability Testing: Internal consistency was assessed using Cronbach’s α coefficient and split-half reliability.
4. Structural equation modeling (SEM) for hypothesis testing.

## 4 Results

### 4.1 Descriptive statistics and correlation analysis

Descriptive statistics were computed for each dimension to describe the central tendency and dispersion of the data. The results are shown in Table 1.

**Table 1:** Descriptive statistics for each dimension.

Dimension	No. of items	Mean	SD	Skewness range	Kurtosis range
Teacher Care	5	4.91	1.43	−0.64–−0.31	−0.58–−0.10
Teacher Clarity	5	4.85	1.37	−0.52–−0.27	−0.39–0.05
Teacher-Student Closeness	5	5.04	1.32	−0.42–−0.16	−0.67–−0.30
Teacher Confirmation	8	4.98	1.41	−0.63–−0.24	−0.52–−0.23
Teacher Credibility–Competence	3	4.91	1.48	−0.58–−0.25	−0.72–−0.33
Teacher Credibility–Goodwill	3	5.00	1.47	−0.68–−0.37	−0.49–−0.38
Teacher Credibility–Trustworthiness	3	5.15	1.44	−0.77–−0.45	−0.55–0.02
Perceived Course Impact	7	5.10	1.38	−0.69–−0.35	−0.53–0.26
Perceived Course Meaningfulness	5	5.26	1.36	−1.02–−0.49	−0.38–0.68
Perceived Course Competence	5	5.08	1.38	−0.83–−0.45	−0.31–0.40

Skewness and kurtosis ranges across items within each dimension; all absolute values are within acceptable limits for normality.

According to Table 1, the scores for each variable were relatively concentrated, with means ranging from 4.57 to 5.48, indicating generally positive perceptions across all dimensions. In terms of data distribution, the absolute skewness of all items is less than 3, and the absolute kurtosis is less than 10, suggesting that the data approximately meet the normality assumption for subsequent multivariate analyses.

### 4.2 Common method bias test

Given the self-report nature of all measures, Harman’s single-factor test was conducted (Podsakoff et al., 2003). An unrotated exploratory factor analysis was performed on all 49 items. As shown in Table 2, the first unrotated factor accounted for 35.09% of the total variance, below the recommended 40% threshold, suggesting that common method bias does not pose a significant threat.

**Table 2:** Harman’s single-factor test result.

Factor	Eigenvalue	% of variance	Cumulative %
1	17.192	35.087	35.087

Having confirmed that common method bias is not a serious concern, this study proceeded to assess the reliability and validity of the scale.

### 4.3 Reliability analysis

In this study, the Cronbach’s α coefficients for all variables ranged from 0.865 to 0.926, all exceeding the acceptable threshold of 0.70. Particularly, Teacher Confirmation showed an α of 0.926, indicating exceptionally high internal consistency for this construct in the Chinese context. The corrected item-total correlation (CITC) for each item exceeded 0.68, well above the 0.40 benchmark. Deleting any item did not substantially increase the α coefficient, suggesting all items contribute meaningfully to their respective scales. Reliability analysis is conducted on the formal research data (N = 446), and the results are shown in Table 3.

**Table 3:** Reliability analysis results (*N* = 446).

Variable	No. of items	Cronbach’s α	CITC range
Teacher Care	5	0.886	0.688–0.754
Teacher Clarity	5	0.874	0.686–0.719
Teacher-Student Closeness	5	0.898	0.706–0.781
Teacher Confirmation	8	0.926	0.716–0.766
Teacher Credibility - Competence	3	0.893	0.778–0.797
Teacher Credibility - Goodwill	3	0.865	0.691–0.781
Teacher Credibility - Trustworthiness	3	0.883	0.759–0.789
Perceived Course Impact	7	0.912	0.707–0.761
Perceived Course Meaningfulness	5	0.880	0.682–0.736
Perceived Course Competence	5	0.907	0.745–0.779

### 4.4 Validity analysis

#### 4.4.1 Exploratory factor analysis

An exploratory factor analysis (EFA) was conducted on the first half of the sample. The KMO value was 0.949, and Bartlett’s test of sphericity was significant (χ^2^ = 14568.571, df = 1,176, *p* < 0.001), indicating suitability for factor analysis. Principal component analysis with varimax rotation extracted 10 factors with eigenvalues greater than 1, explaining 71.30% of the total variance. All factor loadings exceeded 0.58, supporting the construct validity of the adapted scale.

Table 4 presents the simplified rotated component matrix with factor loadings for each set of items.

**Table 4:** Rotated component matrix.

Factor	Items	Loading range
Teacher Care	Q1–Q5	0.724–0.800
Teacher Clarity	Q6–Q10	0.687–0.745
Teacher-Student Closeness	Q11–Q15	0.722–0.800
Teacher Confirmation	Q16–Q23	0.707–0.817
Teacher Credibility-Competence	Q24–Q26	0.804–0.831
Teacher Credibility-Goodwill	Q27–Q29	0.767–0.834
Teacher Credibility-Trustworthiness	Q30–Q32	0.761–0.836
Perceived Course Impact	Q33–Q39	0.585–0.742
Perceived Course Meaningfulness	Q40–Q44	0.688–0.739
Perceived Course Competence	Q45–Q49	0.726–0.772

As can be seen in Table 4, all loadings exceeded 0.58, well above the recommended threshold 0.50 (Hair et al., 2010), with no significant cross-loadings (>0.40). These results support the construct validity of the adapted scale. The clear factor structure lays a solid foundation for further exploring potential variables behind the data, constructing reasonable theoretical models, and conducting more in-depth empirical research. It ensures that the research is based on reliable data structures and enhances the scientific rigor of the research conclusions.

#### 4.4.2 Confirmatory factor analysis

CFA was performed using AMOS 24.0. The model comprised seven first-order latent variables (Teacher Care, Teacher Clarity, Teacher-Student Closeness, Teacher Confirmation, Perceived Course Impact, Perceived Course Meaningfulness, Perceived Course Competence) and one second-order variable (Teacher Credibility) with three sub-dimensions (Competence, Goodwill, Trustworthiness). Each first-order variable was measured by 5, 5, 5, 8, 7, 5, and 5 items respectively; the three sub-dimensions of Teacher Credibility were each measured by 3 items. The decision to model credibility as a second-order factor was theoretically grounded in McCroskey and Teven's (1999) conceptualization, which posits that competence, goodwill, and trustworthiness are inseparable facets of a single overarching construct—source credibility. This theoretical position was further supported by the high inter-correlations among the three sub-dimensions (ranging from 0.49 to 0.66, see Table 5), which would otherwise pose multicollinearity concerns if they were entered as separate predictors. The second-order model also yielded satisfactory fit indices (CFI = 0.970, RMSEA = 0.029), providing empirical support for the hierarchical structure.

**Table 5:** Pearson correlation coefficient matrix between variables.

Variable	1	2	3	4	5	6	7	8
Teacher Care	1							
Teacher Clarity	0.440**	1						
Teacher-Student Closeness	0.412**	0.463**	1					
Teacher Confirmation	0.341**	0.429**	0.367**	1				
Teacher Credibility	0.426**	0.500**	0.467**	0.395**	1			
Perceived Course Impact	0.491**	0.576**	0.502**	0.483**	0.535**	1		
Perceived Course Meaningfulness	0.536**	0.496**	0.474**	0.423**	0.474**	0.533**	1	
Perceived Course Competence	0.442**	0.525**	0.469**	0.455**	0.457**	0.585**	0.478**	1

Numbers 1–8 correspond to the variables listed in the left column, *p < 0.05, **p < 0.01.

*Model fit*. As shown in Table 6, the measurement model demonstrated good fit: χ^2^ /df = 1.381, RMSEA = 0.029, IFI = 0.970, TLI = 0.968, CFI = 0.970, PNFI = 0.839. All indices met or exceeded the recommended thresholds (χ^2^ /df < 3, RMSEA < 0.05, CFI/TLI/IFI > 0.90, PNFI > 0.50), indicating that the model adequately represents the data.

**Table 6:** Model fit indicators (CFA).

Indicator	χ^2^	df	χ^2^/df	RMSEA	IFI	TLI	CFI	PNFI
Optimal Indicator			<3	<0.05	>0.90	>0.90	>0.90	>0.50
Measurement Result	1513.583	1,096	1.381	0.029	0.970	0.968	0.970	0.839

*Convergent validity*. Convergent validity was evaluated by examining whether CR exceeded 0.70 and AVE exceeded 0.50. Table 7 summarizes the composite reliability (CR) and average variance extracted (AVE) for each construct. All CR values exceeded 0.86, well above the 0.70 benchmark, and all AVE values were above 0.58, exceeding the 0.50 threshold. Standardized factor loadings ranged from 0.727 to 0.875 and were all significant at *p* < 0.001. These results support the convergent validity of the scale.

**Table 7:** Convergence validity of confirmatory factor analysis.

Latent variable	CR	AVE	Std. factor loading range
Teacher Care	0.886	0.608	0.727–0.823
Teacher Clarity	0.874	0.582	0.744–0.794
Teacher-Student Closeness	0.899	0.640	0.753–0.835
Teacher Confirmation	0.927	0.613	0.750–0.801
Teacher Credibility–Competence	0.893	0.736	0.840–0.869
Teacher Credibility–Goodwill	0.867	0.686	0.759–0.875
Teacher Credibility–Trustworthiness	0.883	0.716	0.842–0.853
Perceived Course Impact	0.912	0.598	0.763–0.796
Perceived Course Meaningfulness	0.881	0.596	0.730–0.793
Perceived Course Competence	0.908	0.662	0.790–0.825

All factor loadings are significant at *p* < 0.001. CR, Composite Reliability; AVE, Average Variance Extracted.

*Discriminant validity*. Discriminant validity was assessed using the Fornell-Larcker criterion (Fornell and Larcker, 1981), which compares the square root of the AVE of each construct with its correlations with other constructs (see Table 8). As shown in Table 8, the square root of AVE for each construct (bold diagonal) was larger than the inter-construct correlations (off-diagonal entries), confirming good discriminant validity.

**Table 8:** Discriminant validity (Fornell-Larcker).

Variable	1	2	3	4	5	6	7	8
Teacher Care	**0.780**							
Teacher Clarity	0.507***	**0.763**						
Teacher-Student Closeness	0.465***	0.521***	**0.800**					
Teacher Confirmation	0.376***	0.476***	0.400***	**0.783**				
Teacher Credibility	0.537***	0.636***	0.577***	0.487***	**0.719**			
Perceived Course Impact	0.545***	0.644***	0.551***	0.520***	0.660***	**0.774**		
Perceived Course Meaningfulness	0.613***	0.566***	0.531***	0.468***	0.594***	0.591***	**0.772**	
Perceived Course Competence	0.501***	0.590***	0.520***	0.490***	0.565***	0.640***	0.535***	**0.814**

The bold black numbers on the diagonal are the square roots of AVE; *p < 0.05, **p < 0.01, ***p < 0.001; Numbers 1–8 correspond to the variables listed in the left column.

To further examine discriminant validity, the heterotrait-monotrait ratio (HTMT) was calculated. HTMT represents the ratio of the average correlations between items across different constructs to the geometric mean of the average correlations between items within the same construct (Henseler et al., 2015). A threshold of 0.85 is generally recommended, although 0.90 may be acceptable for conceptually similar constructs. As shown in Table 9, all HTMT values ranged from 0.377 to 0.659, below the conservative threshold of 0.85. The largest HTMT value was 0.659, observed between teacher credibility and perceived course impact. These findings indicate satisfactory discriminant validity and are consistent with the Fornell-Larcker criterion reported in Table 8.

**Table 9:** Heterotrait-Monotrait Ratio (HTMT) of discriminant validity.

Variable	1	2	3	4	5	6	7	8
Teacher Care								
Teacher Clarity	0.499							
Teacher-Student Closeness	0.463	0.522						
Teacher Confirmation	0.377	0.478	0.402					
Teacher Credibility	0.534	0.630	0.581	0.486				
Perceived Course Impact	0.545	0.644	0.556	0.525	0.659			
Perceived Course Meaningfulness	0.608	0.566	0.533	0.469	0.596	0.594		
Perceived Course Competence	0.492	0.589	0.520	0.496	0.564	0.642	0.534	

All HTMT values are below 0.85, supporting discriminant validity; Numbers 1–8 correspond to the variables listed in the left column.

*Correlation Analysis*: Pearson correlations were computed to examine the bivariate relationships among the study variables. The results of the correlation analysis are shown in Table 5.

As shown in Table 5, all five teacher interpersonal behaviors were significantly and positively correlated with the three empowerment dimensions (ps < 0.01); perceived course impact correlated with the five teacher behaviors at r = 0.483–0.576, perceived course meaningfulness at r = 0.423–0.536, and perceived course competence at r = 0.442–0.525, providing preliminary support for the hypothesized relationships.

### 4.5 Structural equation model analysis

Structural equation modeling was conducted using AMOS 24.0 to test the hypothesized paths. The structural model demonstrated good fit: χ^2^ /df = 1.401, RMSEA = 0.030, CFI = 0.971, TLI = 0.969, IFI = 0.972, PNFI = 0.839 (similar to the measurement model fit reported in Table 6).

As shown in Figure 1, teacher credibility had the strongest effect on perceived course impact (β = 0.296, *p* < 0.001), teacher care on perceived course meaningfulness (β = 0.320, *p* < 0.001), and teacher clarity on perceived course competence (β = 0.242, *p* < 0.001). Detailed path coefficients and significance levels are reported in Table 10.

**Table 10:** Model path results.

Path	Unstd. estimate	Std. estimate (β)	*p*
Teacher Care → Perceived Course Impact	0.142	0.150	0.003
Teacher Clarity → Perceived Course Impact	0.245	0.251	***
Teacher-Student Closeness → Perceived Course Impact	0.118	0.121	0.018
Teacher Confirmation → Perceived Course Impact	0.150	0.155	***
Teacher Credibility → Perceived Course Impact	0.344	0.296	***
Teacher Care → Perceived Course Meaningfulness	0.305	0.320	***
Teacher Clarity → Perceived Course Meaningfulness	0.145	0.148	0.019
Teacher-Student Closeness → Perceived Course Meaningfulness	0.137	0.140	0.011
Teacher Confirmation → Perceived Course Meaningfulness	0.122	0.125	0.011
Teacher Credibility → Perceived Course Meaningfulness	0.238	0.203	0.009
Teacher Care → Perceived Course Competence	0.152	0.147	0.006
Teacher Clarity → Perceived Course Competence	0.258	0.242	***
Teacher-Student Closeness → Perceived Course Competence	0.158	0.148	0.007
Teacher Confirmation → Perceived Course Competence	0.179	0.169	***
Teacher Credibility → Perceived Course Competence	0.250	0.196	0.012

*p < 0.05, **p < 0.01, **^*^p < 0.001.

Table 10 presents the path coefficients for the relationships between the five teacher behaviors and the three empowerment dimensions.

As shown in Table 10, all five teacher behaviors significantly predicted the three empowerment dimensions (*p* < 0.05), but their relative importance differed across dimensions.

For Perceived Course Impact, teacher credibility had the strongest effect (β = 0.296, *p* < 0.01). This pattern suggests that Chinese students’ trust in teachers’ professional competence, sense of responsibility, and sincere care constitutes the most critical determinant for enhancing their sense of autonomy and control in the course. Teaching clarity also effectively reduce the ambiguity of Chinese students’ academic tasks and strengthen the perceived course impact. In contrast, teacher-student rapport exerted the weakest influence (β = 0.121, *p* = 0.018). This phenomenon may be attributed to the fact that when completing writing-related tasks, Chinese students attach greater importance to teacher authority than to intimate relationships.

For Perceived Course Meaningfulness, teacher care was the strongest predictor (β = 0.320, *p* < 0.001). Teaching clarity had the weakest effect (β = 0.148, *p* = 0.019). These findings indicate that teachers’ care plays a remarkable role in motivating Chinese students to internalize the course value and stimulating their learning motivation. And emotional connection and trust foster internalization of course value more effectively than instructional clarity, affecting Chinese students’ perceived course meaning. Whereas teaching clarity, as an external influencing factor, has a relatively limited effect on Chinese students’ internal recognition and value perception.

For Perceived Course Competence, teaching clarity exerted the strongest influence (β = 0.242, *p* < 0.001). Teacher care showed the weakest effect (β = 0.147, *p* = 0.006), implying that a clear teaching structure and content have a stronger explanatory power for course competence, and students rely more on clear instruction and credible authority than on affective support to build writing confidence. Besides, teachers’ trustworthiness and recognition can further strengthen Chinese students’ perceived competence and enhance their course competence, and regarding course competence, Chinese students place greater emphasis on teachers’ teaching authority and instructional guidance behaviors.

Overall, teacher credibility demonstrated the most consistent predictive power across all three empowerment dimensions (β ranging from 0.196 to 0.296). These results suggest that credibility—rooted in competence, responsibility, and genuine care—serves as the core mechanism of psychological empowerment in high-stakes academic writing contexts.

## 5 Discussion

### 5.1 Differentiated pathways from 5C teacher behaviors to three empowerment dimensions: from “overall effect” to “precise mapping”

The path analysis of this study reveals a nuanced and theoretically novel pattern: for high-cognitive loaded courses, teacher credibility most strongly predicts perceived course impact, teacher care most strongly predicts perceived course meaningfulness, and teacher clarity most strongly predicts perceived course competence.

This study found that teacher credibility, especially its competence and responsibility dimensions, was the strongest predictor of perceived course impact (β = 0.296). This result can be explained from the perspective of vicarious experience in social cognitive theory. When students perceive their teacher as professionally competent and highly responsible, they view the teacher as a “credible model” and an “effective support system.” Consequently, they become more willing to exercise influence in the course—including proposing their own academic ideas, negotiating writing directions with the teacher, and attempting innovative argumentation. Conversely, when teacher credibility is low, students may refrain from expressing their thoughts even when they have them, thereby reducing their perceived course impact.

In this study, teacher care emerged as the strongest predictor of perceived course meaningfulness (β = 0.320). This finding aligns perfectly with the relatedness need pathway in self-determination theory (Deci and Ryan, 2000): care satisfies students’ sense of belonging and being valued, which helps internalize what might otherwise be perceived as an “externally imposed task” into a personally meaningful goal. When teachers express care through encouragement, attention to individual differences, and emotional support, students shift their perception of thesis writing from “doing it for graduation” to “doing it for my own academic growth.” This is highly consistent with a typical statement from the qualitative data of Derakhshan (2022): “The teacher’s encouragement makes me feel that thesis writing is valuable.”

Teacher clarity was the strongest predictor of perceived course competence (β = 0.242). This finding directly supports the enactive mastery experience and verbal persuasion pathways in social cognitive theory (Bandura, 1997). Clear instruction— including explicit task decomposition, transparent evaluation criteria, well-illustrated examples, and timely feedback—helps students accumulate small successes in the high-cognitive-load task of thesis writing (e.g., completing a first draft of the literature review, formulating a clear thesis statement). These small successes gradually build students’ confidence in their writing ability. In contrast, care alone (“I believe in you”) or credibility alone (“I am very experienced”) has limited effects on perceived competence if not translated into actionable, clear guidance.

The differentiated pathways described above indicate that empowerment is not a unitary psychological state but a composite construct consisting of three relatively independent psychological resources: perceived impact, meaningfulness, and competence. Different teacher behaviors activate different resources through different psychological mechanisms. This finding has direct pedagogical implications for thesis writing instruction: to encourage students to actively participate in course decisions (impact), teachers should prioritize building a credible image; to help students internalize the value of the writing task (meaningfulness), teachers should express genuine care; to enhance students’ writing confidence (competence), teachers should provide clear instructional guidance.

### 5.2 Why teacher credibility emerges as the strongest predictor in high-stakes thesis writing contexts

The above findings do not challenge Derakhshan’s (2022) results but rather extend them by demonstrating that the relative salience of 5Cs dimensions shifts systematically across contexts—a context-dependency that enriches rather than contradicts the original framework. In Derakhshan’s (2022) Iranian EFL classrooms, teacher confirmation was the strongest predictor (β = 0.29, higher than credibility’s β = 0.14). In the present study, conducted in Chinese English major thesis writing courses, teacher credibility had the largest standardized path coefficients across the three empowerment dimensions (β ranging from 0.196 to 0.296). This difference in relative statistical associations may be related to contextual variation in educational culture, task demands, and students’ psychological priorities. These paths should be understood as statistical associations within the present cross-sectional model rather than causal effects.

1. Possible Cultural Interpretation. One plausible explanation for the stronger role of credibility in the present study may relate to Chinese educational culture, which is often characterized by high power distance and a tradition of respecting teachers (Hofstede, 2001). Zheng (2021) reported that in Chinese EMI classrooms, teacher credibility predicted student engagement (β = 0.23), offering some indirect support. Pishghadam et al. (2021) also found cross-cultural differences in perceptions of teacher credibility. In high-stakes thesis writing, students may be particularly concerned with whether the teacher is knowledgeable (competence), responsible (duty), and genuinely invested in their success (goodwill). Thus, credibility may co-occur with a greater willingness among students to rely on the teacher’s guidance. Confirmation behaviors, while important, may show weaker unique associations in this sample because they may be perceived as part of the teacher’s expected role.
2. Task Characteristics. In general EFL courses focusing on language skills and content, teacher-student interaction is relatively frequent, and the evaluative stakes are generally lower than those of a high-stakes thesis writing task. The present study focuses on Chinese English major undergraduates’ thesis writing courses, which impose high cognitive load (Sweller, 1988), prolonged engagement, and direct consequences for graduation and degree attainment. The main difficulties students face may include confusion in topic selection, problems in literature synthesis, disorganized argumentation, and uncertainty about academic conventions. These task features may make students more likely to value the teacher’s professional judgment and to perceive the teacher’s guidance as potentially helpful for substantive improvement. Hence, credibility—especially the competence dimension—may show stronger statistical associations with empowerment in this context.
3. Students’ Psychological Needs. According to self-determination theory (Deci and Ryan, 2000), the satisfaction of three basic psychological needs—autonomy, competence, and relatedness—promotes intrinsic motivation, and also students’ prioritization of competence over relatedness may differ across contexts. In Derakhshan (2022) EFL classroom context, characterized by frequent interaction and lower evaluative pressure, students’ primary desire may have been for relatedness (i.e., being recognized and valued by the teacher). Consequently, confirmation behaviors may have been most strongly associated with empowerment. In the present high-stakes thesis writing context study, students face dual pressures of competence anxiety and outcome uncertainty, potentially prioritizing competence assurance and outcome attainment. Their most urgent needs shift towards competence and degree gaining, i.e., questions like “Can I write an acceptable thesis?” The competence dimension of teacher credibility directly addresses the need for competence, reassuring students of the teacher’s capability. More importantly, by demonstrating goodwill and responsibility, a credible teacher creates a supportive yet structured environment—one that provides clear guidance, standards, and feedback. This scaffolded environment alleviated students thesis writing pressure, increasing their probability to their smooth graduation. Accordingly, in the present sample, credibility—rather than confirmation—may have shown the strongest statistical association with empowerment. This explanation is necessarily tentative.

This study carries important theoretical implications: the predictive pattern of the 5Cs scale may be context-dependent—the relative weights of its dimensions may vary across different educational cultures and course types. For Chinese English major students’ thesis writing courses, teachers may consider prioritizing the building a credible image (demonstrating professional competence, fulfilling responsibilities, and expressing genuine care), and then supplementing it with confirmation and clarity behaviors to support student empowerment.

Furthermore, teacher-related variables have been found to explain more variance in foreign language enjoyment than in foreign language classroom anxiety (Dewaele et al., 2018), suggesting that teachers are more effective at fostering positive emotions than at reducing negative ones. In the context of thesis writing, where students’ primary need is for competence assurance (i.e., positive belief in their ability to succeed), teacher credibility—which directly enhances such positive beliefs—may therefore show a stronger statistical association with empowerment than other teacher behaviors aimed primarily at alleviating anxiety. Overall, the present findings indicate statistical associations rather than demonstrated causal effects; future longitudinal and experimental research is needed to test the direction, stability, and generalizability of these pathways.

## 6 Conclusion

### 6.1 Summary of major findings

This study localized the 5Cs Scale for Chinese English major undergraduates and investigated how positive teacher interpersonal behaviors empower students in high-cognitive-load thesis writing courses. Based on 446 third-year English majors from two universities in China, the major findings are:

1. The adapted Chinese 5Cs Scale (49 items) demonstrated sound psychometric properties: KMO = 0.949, cumulative variance = 71.30%, Cronbach’s α > 0.86, CFA fit good (χ^2^ /df = 1.381, RMSEA = 0.029, CFI = 0.970), and both convergent and discriminant validity established.
2. Differentiated predictive pattern emerged: teacher credibility→perceived course impact (β = 0.296); teacher care→perceived course meaningfulness (β = 0.320); teacher clarity→perceived course competence (β = 0.242).
3. Compared to Derakhshan (2022) in Iranian EFL general classrooms (where confirmation was strongest, β = 0.29; credibility weakest, β = 0.14), the present study found a different predictive pattern: credibility became the strongest predictor. This reversal reflects cultural (high power distance in China), task (high-stakes thesis writing vs. Low- stakes EFL courses), and psychological (shift from relatedness to competence assurance) differences.

### 6.2 Theoretical contributions

This study makes three theoretical contributions.

First, it extends the application of positive psychology and the rhetorical/ relational goal theory to a previously underexplored context—high-cognitive-load, high-stakes thesis writing. The findings demonstrate that the relative weight of the 5Cs dimensions is context-dependent, challenging a one-size-fits-all view of teacher interpersonal behaviors.

Second, by revealing differentiated pathways from specific teacher behaviors to specific empowerment dimensions, the study refines the conceptualization of learner empowerment. Rather than treating empowerment as a monolithic construct, it shows that impact, meaningfulness, and competence are distinct psychological resources that can be independently fostered through credibility, care, and clarity, respectively.

Third, the cross-cultural comparison with Derakhshan (2022) provides empirical evidence for the moderating role of educational culture and task type on the 5Cs empowerment relationship, thereby enriching the rhetorical/relational goal theory with a contextualized understanding.

### 6.3 Pedagogical implications

The findings offer actionable guidance for thesis writing instructors in Chinese universities—including both course instructors and one-on-one thesis supervisors—which can be considered from two perspectives: what teachers can do individually and what institutions can do to support them.

For individual teachers, three prioritized strategies emerge. First, to enhance students’ sense of course impact—such as taking initiative, voicing academic opinions, and negotiating writing directions—teachers should prioritize building a credible image. This means demonstrating professional competence, fulfilling responsibilities (e.g., providing timely feedback, keeping promises, addressing student’s concerns) and showing genuine goodwill. Credibility provides students with the confidence to “entrust” their high-stakes thesis to the teacher’s guidance. Second, to foster perceived meaningfulness, i.e., helping students internalize the value of thesis writing, teachers should express genuine care through encouragement, attention to individual difficulties, emotional support, and connecting writing tasks to students’ future academic or career goals. Care helps transform an externally imposed requirement into a personally meaningful endeavor. Third, to boost perceived competence (or writing self-efficacy), teachers should provide clear instructional guidance: explicit task decomposition, transparent evaluation rubrics, well-illustrated examples, and timely, actionable feedback. Clarity reduces cognitive load and enables students to accumulate small successes, gradually building writing confidence. Importantly, these three teacher behaviors are not mutually exclusive; effective teachers should integrate credibility, care, and clarity, adjusting the relative emphasis according to the primary empowerment goal. In Chinese thesis writing contexts, credibility appears to be the most foundational dimension.

Crucially, these three strategies are not meant to be enacted simultaneously at full strength in every teaching episode. Teaching resources—including time, attention, emotional energy, and professional capacity—are inherently finite. It is neither realistic nor sustainable for teachers to constantly demonstrate the highest levels of credibility, care, and clarity all at once across all students and all stages of thesis writing. Recognizing this reality, the differentiated pathways offer a resource-aware, professionally sound practical rule: teachers can identify the most pressing need of their students at a given phase of thesis writing and prioritize the corresponding teacher behavior. For example, at the proposal stage when students feel anxious about topic selection, focusing on credibility (being authoritative and trustworthy) may yield the greatest benefit for student empowerment; later, when students struggle to see the value of their work, shifting emphasis toward care becomes more efficient. This “targeted emphasis’ approach—rather than a one-size-fits-all or “everything at once” ideal—is not a sign of deficiency. On the contrary, it reflects professional judgment and strategic resource allocation, making positive psychology principles feasible and sustainable in real-world teaching.

For universities and institutions, it is essential to recognize that building and sustaining teacher credibility requires more than individual effort. Credibility—characterized by competence, responsibility, and goodwill—depends partly on teachers’ own psychological well-being and work engagement. When teachers feel valued, supported, and psychologically healthy, they are more likely to demonstrate the patience, attentiveness, and expertise that underpin credibility. Therefore, universities should not only train teachers in instructional strategies but also foster a supportive institutional environment that prioritizes faculty well-being, reduces burnout risks, and encourages sustained work engagement (Wang et al., 2022). Such an environment enables teachers to remain professionally committed and emotionally available, which in turn strengthens the trust that students place in their guidance.

While this study focused on thesis writing courses, the same differentiated principles apply to one-on-one thesis supervision, where supervisors likewise need to balance credibility, care, and clarity according to students’ most pressing needs.

### 6.4 Limitations and future directions

Several limitations should be acknowledged. First, the cross-sectional design precludes causal inferences; future studies may adopt longitudinal or experimental designs to establish causality. Second, the sample was drawn from two comprehensive universities, which may limit generalizability to other institutional types, e.g., foreign language specialty institutions, or vocational colleges. Third, all measures were self-reported, and although common method bias was not a serious concern, future research could incorporate behavioral indicators, e.g., actual writing performance, revision quality or teacher-reported data. Fourth, the study did not include qualitative data; future research could use interviews or open-ended questionnaires to capture students’ nuanced perceptions of teacher credibility, care, and clarity.

Future research may also extend the present work in several directions: (a) cross-cultural comparative designs that directly test the moderating role of cultural dimensions (e.g., power distance, uncertainty avoidance) on the 5Cs predictive pattern; (b) examining whether the same differentiated pathways hold in other high-stakes academic writing contexts (e.g., master’s theses or doctoral dissertations); (c) exploring potential interaction or mediation effects among the 5Cs dimensions (e.g., whether credibility enhances the effect of clarity on competence); and (d) investigating how AI tools (e.g., ChatGPT, DeepSeek) reshape students’ perceptions of teacher credibility and the relative importance of different teacher behaviors, as well as whether teacher credibility should be expanded to include an AI literacy dimension—namely, the capacity to guide students in the critical use and evaluation of AI tools. Because AI use was not measured in this study, these remain speculative directions for future inquiry.

### 6.5 Concluding remarks

In conclusion, this study successfully localized the 5Cs Scale for Chinese English major undergraduates and provided empirical evidence that, in high-cognitive-load thesis writing courses, teacher credibility emerges as the most critical predictor of student empowerment, followed by teacher care and teacher clarity for different empowerment dimensions. The reversal of the prediction order compared with Derakhshan (2022) underscores the context-dependent nature of positive teacher interpersonal behaviors. For thesis writing instruction in China, building a credible teacher image—characterized by competence, responsibility, and goodwill—should be the first priority, complemented by care and clarity to maximize students’ psychological empowerment, writing confidence, and ultimately, their academic success.

## Data Availability

The original contributions presented in the study are included in the article/Supplementary material, further inquiries can be directed to the corresponding author.
