# Supplementary material for: Localizing the 5Cs Scale in China: when teacher credibility outperforms confirmation in high-stakes thesis writing
Source: Front Psychol. 2026 Oct 20;17:1955402. doi: 10.3389/fpsyg.2026.1955402 (PMC13727553; doi:10.3389/fpsyg.2026.1955402)
Supplement: Supplementary file 1 [file Supplementary_file_1.docx]

**Appendix A:** Final version of the Localized 5Cs Scale

Teacher Care

1. My teacher adopts my views on thesis writing in class.

2. My teacher provides feedback on my proposal draft.

3. My teacher responds to my messages on Xuexitong, WeChat, email, etc.

4. My teacher answers my questions carefully.

5. My teacher encourages me when I share my ideas.

Teacher Clarity

6. My teacher clearly defines core concepts.

7. My teacher’s teaching objectives are clear and explicit.

8. My teacher’s instruction is straightforward and to the point.

9. My teacher uses clear and relevant examples.

10. My teacher provides specific and explicit instructional guidance.

Teacher-Student Closeness

11. My relationship with my teacher is: bad – good

12. My relationship with my teacher is: cold – warm

13. My relationship with my teacher is: distant – close

14. My relationship with my teacher is: superficial – deep

15. My relationship with my teacher is: uncomfortable – comfortable

Teacher Confirmation

16. My teacher values my questions and viewpoints in class.

17. My teacher provides specific feedback (oral/written) on assignments.

18. My teacher maintains eye contact during instruction.

19. My teacher uses an interactive teaching style.

20. My teacher displays positive emotions toward the class.

21. My teacher conveys confidence in my learning ability.

22. My teacher employs diverse teaching strategies.

23. My teacher adjusts instruction based on students’ questions.

Teacher Credibility – Competence

24. My teacher’s professionalism: unprofessional – highly professional

25. My teacher’s knowledge: limited – extensive

26. My teacher’s competence: incompetent – fully competent

Teacher Credibility – Goodwill

27. My teacher’s attention to my needs: neglectful – attentive

28. My teacher’s concern for me: indifferent – genuinely cares about my growth

29. My teacher’s disposition: cold and harsh – warm and considerate

Teacher Credibility – Trustworthiness

30. My teacher: unreliable – reliable

31. My teacher: irresponsible – highly responsible

32. My teacher’s treatment of students: biased and unfair – just and fair

Perceived Course Impact

33. I can choose my own learning methods.

34. This course encourages innovative learning strategies.

35. I can participate in important learning decisions.

36. I can create a supportive learning environment.

37. I can decide how to complete tasks.

38. I can facilitate the learning process of the course.

39. I can influence the teacher’s instructional decisions.

Perceived Course Meaningfulness

40. The course content is valuable to me.

41. The learning objectives match my needs.

42. This course is inspiring.

43. The course tasks are of great value to me.

44. This course helps me achieve my future goals.

Perceived Course Competence

45. I possess the skills necessary for success in this course.

46. I believe I can achieve the course objectives.

47. I am confident that I can do well in this course.

48. I have the essential qualities for success in this course.

49. I feel at ease and capable in this course.

Note: (1) “R” indicates a reverse-coded item; (2) Item 9, 17, 40 were deleted for low factory loading; (3) Xuexitong is a digital course platform for Chinese students.

**Appendix B**: Item Mapping of the Adapted 5Cs Scale

This appendix provides a transparent record of the scale adaptation process. The original instrument (Derakhshan, 2022) comprised 102 items relevant to this study (67 5Cs items + 35 Learner Empowerment items). Through expert review, cultural adaptation, and pilot testing, 50 items were deleted, and several items were revised to fit the Chinese thesis writing context. The final scale retained 49 items. Deletion criteria included: (a) cultural inappropriateness for the Chinese educational context; (b) substantive semantic overlap with other items in the same subscale; (c) contextual irrelevance to thesis writing courses; and (d) low factor loadings (< 0.50) or poor discrimination indices identified in the pilot study.

**Teacher Care Scale**

| **Original Item** | **Final Item** | **Action** | **Rationale** |
| --- | --- | --- | --- |
| 2. My teacher encourages me. | 5 | Revised | Contextualized to thesis writing (sharing ideas) |
| 4. ...uses my knowledge in the class. | 1 | Revised | Contextual adaptation to thesis writing |
| 5. ... checks my homework. | 2 | Revised | Contextual adaptation to thesis writing |
| 9. ... responds to my phone calls and emails. | 3 | Revised | Updated to reflect Chinese digital learning platforms |
| 16. ... carefully answers my questions. | 4 | Revised | Wording refined for clarity |
| 1,3, 6, 7, 8, 10-15, 17, 18 | — | Deleted | Removed due to semantic overlap, cultural inappropriateness (reverse-coded items about favoritism, anger, etc.), or contextual irrelevance |
|  |  |  |  |

**Teacher Clarity Scale**

| **Original Item** | **Final Item** | **Action** | **Rationale** |
| --- | --- | --- | --- |
| 1. My teacher clearly defines major concepts... | 6 | Revised | Shortened for conciseness |
| 5. ...'s objectives for the course are clear. | 7 | Revised | Wording refined for clarity |
| 6. ... is straightforward in his/her... lecture. | 8 | Revised | Wording refined |
| 8. ... uses clear and relevant examples... | 9 | No change | — |
| 10. ... is explicit in her or his instruction. | 10 | Revised | Wording refined for clarity |
| 2, 3, 4, 7, 9 | — | Deleted | Removed due to reverse-coding, semantic overlap, or contextual irrelevance; Item 9 later deleted due to low factor loading |

**Teacher-Student Closeness Scale**

| **Original Item** | **Final Item** | **Action** |
| --- | --- | --- |
| 1. My relationship with my teacher is: good/bad | 11 | No change |
| 2. ... warm/cold | 12 | No change |
| 3. ... close/distant | 13 | No change |
| 4. ... shallow/deep | 14 | No change |
| 5. ... uncomfortable/comfortable | 15 | No change |

**Teacher Confirmation Scale**

| **Original Item** | **Final Item** | **Action** | **Rationale** |
| --- | --- | --- | --- |
| 2. My teacher indicates that he/she appreciates students' questions or comments. | 16 | Revised | Wording refined |
| 5... gives oral or written feedback on students' work. | 17 | Revised | Wording refined |
| 6. ... establishes eye contact during class lectures. | 18 | Revised | Wording refined |
| 7. ... uses an interactive teaching style. | 19 | No change | — |
| 10. ... smiles at the class. | 20 | Revised | Broadened to include more than smiling |
| 11.... communicates that he/she believes that students can do well in the class. | 21 | Revised | Wording refined |
| 13. ...u ses a variety of teaching techniques... | 22 | Revised | Wording simplified |
| 16. ... is willing to deviate slightly from the lecture when students ask questions. |  | Revised | Wording refined |
| 1, 3, 4, 8, 9, 12, 14, 15 | — | Deleted | Removed due to contextual irrelevance or semantic overlap; Item 1 later deleted due to low factor loading |

**Teacher Credibility Scale**

**Competence Sub-dimension**

| **Original Item** | **Final Item** | **Action** | **Rationale** |
| --- | --- | --- | --- |
| 3. My teacher is inexpert-expert | 24 | Revised | Wording adapted to Chinese educational context |
| — | 25 | New | Added to better capture the “knowledge” aspect in Chinese context |
| 5. ... incompetent-competent | 26 | No change | — |
| 1, 2, 4, 6 | — | Deleted | Removed due to cultural inappropriateness (e.g., “bright-stupid”) or semantic overlap |

**Goodwill Sub-dimension**

| **Original Item** | **Final Item** | **Action** | **Rationale** |
| --- | --- | --- | --- |
| 10. My teacher concerned with me-unconcerned with me | 27 | Revised | Wording refined |
| 7. ... cares about me-doesn't care about me | 28 | Revised | Wording refined to specify “growth” in Chinese context |
|  |  |  |  |
| 12. ... not understanding-understanding | 29 | Revised | Wording refined for clarity |
| 8, 9, 11 | — | Deleted | Removed due to semantic overlap with retained items |

**Trustworthiness Sub-dimension**

| **Original Item** | **Final Item** | **Action** | **Rationale** |
| --- | --- | --- | --- |
| 14. My teacher is untrustworthy-trustworthy | 30 | Revised | Wording refined |
| 17. ... unethical-ethical | 31 | Revised | Wording adapted to Chinese context |
| 18. ... phoney-genuine | 32 | Revised | Wording adapted to Chinese context |
| 13, 15, 16 | — | Deleted | Removed due to semantic overlap with retained items |

**Learner Empowerment Scale**

**Impact Sub-dimension**

| **Original Item (Derakhshan, 2022)** | **Final Item** | **Action** | **Rationale** |
| --- | --- | --- | --- |
| 2. I have a choice in the methods I can use to perform my work. | 33 | Revised | Wording refined for clarity |
| 6. Alternative approaches to learning are encouraged in this class. | 34 | Revised | Wording refined |
| 8. I have the opportunity to make important decisions in this class. | 35 | Revised | Wording refined |
| 10. I have the power to create a supportive learning environment in this class. | 36 | Revised | Wording simplified |
| 12. I can determine how tasks can be performed. | 37 | Revised | Wording refined |
| 13. I make a difference in the learning that goes on in this class. | 38 | Revised | Wording refined |
| 15. I can influence the instructor. | 39 | Revised | Wording refined to specify “instructional decisions” |
| 1, 3, 4, 5, 7, 9, 11, 14, 16 | — | Deleted | Removed due to semantic overlap, reverse-coding or low factor loading |

**Meaningfulness Sub-dimension**

| **Original Item** | **Final Item** | **Action** | **Rationale** |
| --- | --- | --- | --- |
| 1. The tasks required of me in this class are personally meaningful. | 40 | Revised | Wording refined to focus on content |
| 2. I look forward to going to this class | 41 | Revised | Wording refined |
| 3. This class is exciting. | 42 | Revised | Wording refined |
| 6. The tasks required of me in this class are valuable to me. | 43 | Revised | Wording refined |
| 8. This course will help me achieve my future goals. | 44 | Revised | Wording refined for clarity |
| 4, 5, 7, 9, 10 | — | Deleted | Reverse-coded items or semantic overlap |

**Competence Sub-dimension**

| **Original Item** | **Final Item** | **Action** | **Rationale** |
| --- | --- | --- | --- |
| 3. I possess the necessary skills to perform successfully in class. | 46 | Revised | Wording refined |
| 5. I believe that I am capable of achieving my goals in this class. | 47 | Revised | Wording refined |
| 6. I have faith in my ability to do well in this class. | 48 | Revised | Wording refined |
| 7. I have the qualifications to succeed in this class. | 49 | Revised | Wording refined |
| 9. I feel very competent in this class. |  | Revised | Wording refined to include “ease” |
| 1, 2, 4, 8 | — | Deleted | Removed due to reverse-coding or semantic overlap |

Note: The final 49-item instrument is presented in Appendix A. Item numbers in the “Final Item” column correspond to Appendix A.
